# Bone-protective effects of deer-hide gelatin in cyclophosphamide-induced osteoporosis rats

**DOI:** 10.3389/fphar.2025.1631924

**Published:** 2025-10-07

**Authors:** Hongyun Mao, Xinyue Zhao, Shaoqin Mo, Haili Wang, Rui Liu, Yu Xie, Yong Huang, Yunfeng Zheng, Yongqing Hua

**Affiliations:** ^1^ Jiangsu Provincial Key Laboratory of Functional Substances in Traditional Chinese Medicine Formulae and Innovative Drug Discovery, Nanjing, China; ^2^ School of Pharmacy, Nanjing University of Chinese Medicine, Nanjing, China; ^3^ Jiangsu Key Laboratory for Pharmacology and Safety Evaluation of Chinese Materia Medica, Nanjing University of Chinese Medicine, Nanjing, China; ^4^ Guizhou Guangjitang Pharmaceutical Co., Ltd., Guiyang, China

**Keywords:** deer-hide gelatin, chemotherapy, cyclophosphamide, osteoporosis, collagen, peptides, network pharmacology

## Abstract

Chemotherapy is a cornerstone of cancer treatment, but its adverse effects, particularly those related to the cardiovascular and skeletal systems, are drawing more attention. According to studies, the PI3K/AKT signaling pathway may be involved in myelosuppression and cardiotoxicity, two types of multi-organ damage caused by chemotherapy. Despite the absence of thorough research, deer hide gelatin (DHG), a traditional Chinese medicine high in collagen, has shown promise in the prevention and treatment of skeletal and hematological disorders. This study aimed to evaluate the protective effects of DHG on chemotherapy-induced osteoporosis (OP) in rat bone tissue, as well as the material basis and mechanisms of its anti-OP activity. The results showed that DHG reversed the decrease in bone mineral density induced by chemotherapy, improved bone biomechanical properties, and ameliorated bone microstructure. DHG promoted the expression of the osteoblast-related indicators BALP and P1NP while suppressing the expression of the osteoclast-related marker TRACP-5b. Protein mass spectrometry screening was used to find putative anti-OP bioactive peptides. According to network pharmacology predictions, the PI3K signaling pathway may be the mechanism by which the active peptides in DHG produce their anti-OP actions. Additionally, immunofluorescence investigation demonstrated that DHG inhibited MMP9 expression while increasing RUNX2 expression. *In vitro* experiments also confirmed that DHG active peptides promoted bone formation by activating the PI3K/AKT/ERK signaling pathway, upregulating RUNX2 protein expression, and promoting osteoblast differentiation and mineralization. In conclusion, DHG has protective benefits against OP caused by chemotherapy. This also raises the possibility that DHG could be useful in the broader management of chemotherapy side effects (e.g., potentially related to cardio-oncology, considering the pathway’s important role in organs like the heart), warranting further investigation.

## 1 Introduction

Malignant tumors are increasing year by year, posing a significant threat to human health. Chemotherapy, as a cornerstone of tumor treatment, has significantly improved patient survival rates, yet focusing on the quality of life for cancer patients is equally crucial (Lewandowska et al., 2020). Chemotherapy-induced side effects are widespread, affecting multiple systems, including the hematopoietic, immune, skeletal, and cardiovascular systems (Wang et al., 2006; Lin et al., 2024; Alizadehasl et al., 2024). These adverse reactions can be attributed to various mechanisms, such as bone marrow suppression (Shao et al., 2013) and inhibition of ovarian function (Kim et al., 2021). Cyclophosphamide (CTX) is a first-line chemotherapy drug and one of the most extensively studied agents. In cancer treatment, CTX can lead to myocardial dysfunction and heart failure, with an incidence rate as high as 7%–28% (Zamorano et al., 2016). However, the mechanisms underlying its cardiotoxic effects remain unclear. Chemotherapy-induced bone loss involves multiple mechanisms, including gonadal dysfunction (Oostra et al., 2015; Kim et al., 2020), direct effects on osteoblasts and osteoclasts (Cameron et al., 2010; Lee et al., 2017), and damage to organs involved in vitamin D metabolism (Kuzu et al., 2019; Peng et al., 2023). CTX can cause bone marrow suppression, leading to decreased hematopoiesis, immune function suppression, and reduced bone formation (Zhao et al., 2017). Furthermore, CTX-induced inhibition of ovarian function leads to hormonal changes (Hirbe et al., 2006; Hadji et al., 2009; Markowska et al., 2024), which may also contribute to the aforementioned adverse reactions. Chemotherapy-induced osteoporosis (OP) is caused by factors such as bone marrow suppression, severely impairing the skeletal health of cancer patients (Yao et al., 2020; Rossi et al., 2022). Clinical observations confirm that chemotherapy increases the risk of bone mineral density (BMD) decline and bone loss (Nisha et al., 2023), leading to fragility fractures and reduced quality of life (Shapiro et al., 2019; Viswanathan et al., 2018).

Interestingly, increasing evidence suggests that specific key signaling pathways are involved in the response of various tissue cells to chemotherapy stress. For example, the PI3K/AKT signaling pathway plays a crucial role in cell survival, growth, differentiation, and stress response in various cell types, including skeletal cells (Sudhakaran et al., 2023) and cardiomyocytes (Deng and Zhou, 2023; Lin et al., 2015). Dysregulation of this pathway is associated with chemotherapy-induced organ damage. The PI3K/AKT/ERK pathway is considered a key regulator of osteoblast differentiation and bone formation (Zhu et al., 2020b; Kim et al., 2014; Ye et al., 2020b; Heo et al., 2018; Ding et al., 2019). Simultaneously, it is also closely related to cardiomyocyte survival and function, playing a critical protective role in resisting cardiomyocyte apoptosis and inhibiting myocardial fibrosis (Ghafouri-Fard et al., 2022). This overlap in crucial regulatory pathways provides a potential molecular link between the skeletal side effects of chemotherapy and effects on other organs (such as the heart), which is relevant to the scope of cardio-oncology, an emerging interdisciplinary field dedicated to studying the impact of cancer treatment on the cardiovascular system and strategies to improve cardiovascular outcomes in cancer patients.

Deer hide gelatin (DHG), derived from the hides of *Cervus elaphus* Linnaeus or *Cervus nippon* Temminck, is a traditional Chinese medicine and a potential functional food rich in collagen protein. With the expansion of artificial breeding, farmed red deer and sika deer have been officially classified as domestic livestock in China (Ministry of Agriculture and Rural Affairs, 2020). The previously limited availability of deer hide resources has been significantly alleviated, sparking growing interest in research on the unique medicinal effects of DHG. Collagen peptides have garnered increasing attention for their efficacy in preventing and treating OP (Noma et al., 2017; Konig et al., 2018; Elisangela and Bernardes, 2016). Collagen peptides from various sources have been shown to benefit bone health by promoting bone formation and reducing bone resorption, thereby increasing bone mineral density (BMD) and enhancing bone structure (Ye et al., 2020a; Chuu et al., 2024; Song et al., 2019). In addition to its effects on bone, collagen derivatives have been reported to possess broader cellular benefits, including antioxidant and anti-inflammatory properties (Hijlkema et al., 2022; Xin et al., 2025), which may be relevant to mitigating chemotherapy-induced organ stress.

According to traditional Chinese medicine literature, deer hide gelatin has the effects of tonifying Qi, nourishing Yin, and blood, strengthening the kidneys and consolidating Jing, as well as strengthening tendons and bones (Wu et al., 2020a; Guizhou Medical Products Administration, 2019). While previous studies have suggested the potential role of DHG in areas such as immunomodulation (Hongyun et al., 2024), experimental evidence regarding its effects on osteoporotic diseases is limited. CTX can induce bone loss by inhibiting sex hormone secretion and reducing the activity of both osteoblasts and osteoclasts, making it a commonly used drug to establish OP models (Hirbe et al., 2006; Hadji et al., 2009; Zhao et al., 2017; Wang et al., 2019). This study aimed to investigate the bone-protective effects of DHG on chemotherapy-induced OP in a rat model using CTX. We will focus on evaluating the impact of DHG on skeletal health and explore its potential molecular mechanisms through techniques such as network pharmacology, serum metabolomics, and *in vitro* cell experiments. By exploring the mechanisms of DHG action, particularly its potential interactions with PI3K/AKT and other signaling pathways identified in network analysis, we aim to understand its bone-protective effects and discuss their relevance within the broader context of managing chemotherapy side effects. Given the critical role of these pathways in multiple organs affected by chemotherapy, the findings of this study may provide initial clues for discovering new targets and therapeutic strategies that can simultaneously act on the skeletal and cardiovascular systems in the context of chemotherapy, aligning with the theme of cardio-oncology. The study results aim to provide new strategies for the prevention and treatment of OP, as well as improve the overall health of cancer patients undergoing chemotherapy.

## 2 Materials and methods

### 2.1 Reagents

The DHG (20180103) used in this study is a commercially available product provided by Guizhou Guangjitang Pharmaceutical Co., Ltd. (Guiyang, China). It is a solid gelatin made by decocting and concentrating dried or fresh hides of *Cervus elaphus* Linnaeus or *Cervus nippon* Temminck of the Cervidae family. Currently, DHG is included in the Standard for Chinese Medicinal and Ethnic Medicinal Decoction Pieces of Guizhou Province (Guizhou Medical Products Administration, 2019). All voucher specimens (No. NJUTCM-20201206) are stored in the herbarium of Nanjing University of Chinese Medicine. The DHG products used in this study were derived from farmed sika deer and red deer, with breeding and processing licenses complying with national laws and regulations. Four peptide biomarkers of DHG were identified by UPLC-MS/MS and MRM, as reported in previous studies (Han et al., 2021; Liu et al., 2019). The CTX (F13IS206786) and Alendronate (AL) (F16HS175560) were purchased from Yuanye Bio-Technology Co., Ltd. (Shanghai, China). The Alizarin Red S (ARS, C0148S) and LY294002 (S1737-1 mg) were purchased from Beyotime Biotechnology (Jiangsu, China). Fetal bovine serum (FBS), penicillin/streptomycin, 0.25% trypsin/EDTA, and α Modified Eagle’s Medium (α-MEM) were obtained from Biological Industries (Kibbutz Beit Haemek, Israel). The peptides of P2 were synthesized by GenScript Biotech Co., Ltd. (C9456PDQG0-11-PE0839, Nanjing, China). The primary antibody against active matrix metalloproteinase-9 (MMP9) was purchased from Proteintech (10375-2-AP, Wuhan, China). The primary antibodies against active runt-related transcription factor 2 (Runx2, AF5186), p-AKT (AF3263), AKT (AF6261), p-ERK (AF1015) and ERK (AF0155) were purchased from Affinity Biosciences (OH, United States). The primary antibody against β-Actin was purchased from ABclonal (AC026, Wuhan, China). The secondary antibody was purchased from Zen-Bio (511203, Chengdu, China). The immunofluorescence staining (TSA) kit (20230915) was purchased from Aifang Biological Co., Ltd. (Hunan, China), including universal secondary antibody fluorescent dyes TYR-520 and TYR-570.

The recommended clinical dose of DHG for human use ranges from 3 to 9 g/d, with an experimental dose set at 6 g/d, equivalent to 0.1 g/kg/d based on a standard body weight of 60 kg. Utilizing the body surface area ratio conversion (Nair and Jacob, 2016), a dose of 0.54 g/kg was calculated for rats weighing approximately 400 g, the estimated average body weight at 4 months of age. To achieve a dose-dependent effect, doses of 0.5, 1, and 2 times the human-equivalent dose were used in the rat model. For oral administration, DHG was solubilized by heating in distilled water.

### 2.2 Animals and establishment of the OP model

Three-month-old Specific Pathogen-Free female Sprague-Dawley rats (*n* = 72), initially weighing 250 ± 20 g, were acquired from Hangzhou Medical College of Zhejiang Province, with the animal certificate No. 20230508Azz010000918. These trial animals were housed at the Experimental Animal Center of Nanjing University of Traditional Chinese Medicine, where they were cared for in strict accordance with the Guide for the Management and Use of Experimental Animals. The animals were provided with 12 h of light and 12 h of darkness to replicate a natural day-night rhythm at a room temperature of 20 °C – 26 °C and a relative humidity of 50%–60%. All procedures involving the animals were conducted with authorization from the Animal Ethics Committee of Nanjing University of Traditional Chinese Medicine under the ethical clearance number 202307A011.

Following a 7-day acclimation period, the rats were randomly assigned to six groups, each with 12 rats per group, stratified by body weight. The groups included a control, CTX, AL (7.35 mg/kg), DHG low dose (0.27 g/kg, DHG-L), DHG medium dose (0.54 g/kg, DHG-M), and DHG high dose (1.08 g/kg, DHG-H). All groups, except the Control, received daily intraperitoneal CTX injections at a dose of 8 mg/kg/day administered in a volume of 1 mL/kg for 35 consecutive days. Rats in the Control and CTX groups were administered normal saline. In contrast, those in the DHG-treated groups received their corresponding DHG doses at 5 mL/kg once daily. The body weight of each rat was determined every 3 days to adjust the dosing.

On the day before the conclusion of the experiment, the rats underwent a 24-h fasting period. The rats were then individually weighed before anesthesia was induced by intraperitoneal injection of 10% ethyl carbamate, administered at a dosage of 1 g/kg. Blood samples were subsequently taken from the abdominal aorta and subjected to centrifugation at 3,000 rpm for 10 min to separate the serum, which was stored at −80 °C for subsequent analysis. For assessment of bone biomechanical properties, the left femur and tibia were individually placed in 10 mL EP tubes filled with normal saline and stored at −80 °C. The right femur and tibia were fixed in EP tubes containing 4% paraformaldehyde (PFA) for 48 h. The right femur and tibia were scanned by micro-computed tomography (Micro-CT) to determine bone quantitative parameters and microstructure. The BMD of the right femur and tibia was determined by dual-energy X-ray absorptiometry.

### 2.3 Symptom score

The changes in symptoms were scored every week, with the scoring criteria for symptom scores shown in Table 1.

**TABLE 1:** Symptom score table.

Score	Symptoms		
	Hair color and condition	Behavioral state	Morphological and mental state
0	Hair is neat, hair color normal and shiny	Agility, straight back, active behavior, strong grip	Nose and lips are clear and moist, pale pink, eyes bright, tail pink, energetic
1	Hair slightly messy, hair color slightly shiny	Agility, activity slightly reduced, grip holding power	Face, ears, tail color pale, slightly dark, poor spirit
2	Hair messy, hair color less luster	Slow movement, wheezing after slight activity, slight grip	Face, ears, tail color white and unadorned, dark and dull, listless
3	Hair messy, unkempt, dull	Slow movement, hunched waist, shortness of breath, weak grip	Face, ears, tail pale and cool, blood red, extremely listless

### 2.4 Behavioral experiments

#### 2.4.1 Running endurance test

Rats were placed on a motorized treadmill apparatus (KW-PT, Calvin Biology, Nanjing, China) for an exercise regimen. The initial treadmill velocity was gradually increased to 0.25 m/s at an acceleration rate of 0.0125 m/s^2^ over 20 s, after which the rats maintained a consistent pace for 5 min. To ensure the rats remained engaged in the exercise, the electric shock feature of the treadmill was activated, with the stimulating current set to 0.1 mA. This safety measure encouraged the rats to maintain pace with the treadmill movement to avoid contact with the electric shock apparatus positioned at the rear of the treadmill. Continuous movement was observed and recorded over the 5-min trial duration. To acclimatize the rats to the treadmill and habituate them to the exercise protocol, a 1-week daily training session was conducted before the initiation of the formal experiment.

#### 2.4.2 Weight-bearing swimming experiment

Rats were subjected to an exhaustive swimming test in a cylindrical tank with a diameter of 31 cm and a water depth of 30 cm, where the temperature was strictly controlled at 30 °C ± 2 °C. Before the formal test, the animals underwent a 3-day acclimatization period consisting of daily 10-min swimming sessions. After the acclimatization phase, a formal loaded swimming test was conducted, where a lead weight corresponding to 10% of the rat’s body weight was attached to its tail. The duration of exhaustive swimming was timed, with the endpoint of exhaustion defined as the rat’s inability to resurface within 8 s of submersion.

### 2.5 Serum analysis

Serum concentrations of bone-specific alkaline phosphatase (BALP), tartrate-resistant acid phosphatase 5b (TRACP-5b) and N-terminal propeptide of type I procollagen (P1NP) were determined employing enzyme-linked immunosorbent assay (ELISA) kits (AF3494-A, AF3282-A, AF40059-A, respectively; AiFang Biological, Hunan, China) as per the manufacturer’s protocols.

### 2.6 BMD and bone mineral content (BMC) assessment

The PFA-fixed right femur and tibia samples were subjected to BMD and BMC analysis using dual-energy X-ray absorptiometry employing a MEDIX90 instrument (MEDLINK, Mauguio, France) set to the small animal mode with fine scanning resolution.

### 2.7 Micro-CT

Trabecular morphometry was assessed by Quantum GX Micro-CT (PerkinElmer, MA, United States), as specified in the system’s application instructions. The right femur was precisely positioned along the longitudinal axis within the sample scanning tube and secured using foam to prevent motion during scanning. Optimal imaging parameters were set, with a voltage at 65 kV and the current calibrated to 384 μA, using a 1-mm filter employed to refine the scan. The resolution was fine-tuned to 18 μm to ensure detailed morphometric analysis. Following scanning, the acquired CT images were reconstructed with the imaging system’s proprietary software Nrecon v1.6, followed by further analysis with CTAn v1.14. For consistent and reproducible measurements, the growth plate was designated as the reference landmark. A series of 100 consecutive CT images immediately below the growth plate were analyzed to evaluate the bone trabeculae.

### 2.8 Biomechanical strength

The left tibia was gradually brought to room temperature before mechanical testing. The biomechanical evaluation was conducted utilizing a three-point bending test to assess the bone’s resistance. This analysis was performed with an INSTRON 3367 electronic universal testing machine (Instron, Boston, United States). The support span (L) was fixed at 20 mm, and a loading rate of 1.2 mm/min was implemented during the testing procedure. Before applying mechanical loading, the cross-sectional dimensions critical for calculating biomechanical parameters were acquired from tibia images obtained by micro-CT scanning. These dimensions were then used to calculate indices including bone maximum load, stress, stiffness coefficient, and elastic modulus, by following established methodologies described in previous studies (Deckard et al., 2017; Fridoni et al., 2015).

### 2.9 Histological examination

A 1.5-cm section from the anterior aspect of the left tibia was excised using surgical-grade scissors and subjected to a decalcification process in 10% ethylenediaminetetraacetic acid for 30 days, then embedded in paraffin wax and subsequently sectioned into slices of 10 μm for microscopic evaluation. To prepare the sections for staining, they were initially deparaffinized in xylene and rehydrated using a descending ethanol gradient. The rehydrated sections were first stained with hematoxylin for 5 min and then rinsed thoroughly under running water. A subsequent clearing step in water was undertaken for 1 min, followed by eosin staining for an additional 5 min, providing a contrast stain for visualizing cellular and extracellular matrix. The stained sections were dehydrated with anhydrous ethanol, cleared with xylene, and permanently mounted with a neutral resin. For the microscopic analysis, the mounted slides were examined using a pathology-specific imaging system Vectra 3.0. Pathological imaging software (Mantra, Perkin Elmer) facilitated the digitization of images acquired during the microscopic investigation.

### 2.10 Immunofluorescence analysis

The paraffin-embedded sections were initially subjected to deparaffinization with xylene, followed by systematic rehydration through a graded ethanol series. The tissue was enzymatically digested with hyaluronidase at a controlled temperature of 37 °C for 30 min, followed by pepsin digestion under identical conditions to expose antigenic sites. To suppress endogenous peroxidase activity, the sections were treated with 3% hydrogen peroxide for 15 min. Non-specific binding was minimized using a 5% bovine serum albumin-blocking solution. The slides were incubated overnight at 4 °C with a rabbit anti-MMP9 primary antibody at a dilution of 1:500.

The immunofluorescence staining was performed using a TSA kit. After rigorous washing with phosphate-buffered saline with Tween 20 (PBST), a universal secondary antibody was applied at room temperature for 30 min. Following a further PBST wash, fluorescence staining was performed using TYR-520 for 10 min. The protocol was followed by the addition of a multiplex immunohistochemistry antibody elution buffer for another 30 min, allowing for serial staining. An additional PBST wash was performed, followed by incubation with a rabbit anti-Runx2 primary antibody at a dilution of 1:500, also overnight at 4 °C. A subsequent application of the universal secondary antibody was performed for 30 min at room temperature, followed by three washes in PBST. The TYR-570 was added for fluorescence staining for 10 min, followed by additional PBST washes.

The 4′-6-diamidino-2-phenylindole (DAPI) staining was conducted in a subdued light environment at room temperature for 10 min to stain nuclear content, followed by a final series of PBST washes. The sections were treated with an anti-fade mounting medium. Microscopic analysis was performed using a BC43 benchtop confocal microscope (ANDOR, Belfast, United Kingdom) with a ×20 magnification objective, providing high-resolution images with detailed tissue morphology and protein expression.

### 2.11 DHG bioactive peptides analysis

Based on the team’s preliminary analysis of the protein mass spectrometry data from DHG (Liu et al., 2019), we conducted a comprehensive examination of its peptide sequence data. The Peptide Spectrum Matches (PSM) value is a crucial parameter in mass spectrometry analysis, representing the number of matched peptide segments within a mass spectrum. In mass spectrometry, a higher PSM value typically correlates with increased confidence in protein identification while simultaneously reflecting the protein’s abundance. Furthermore, in mass spectrometry analysis, the magnitude of the intensity values is intrinsically linked to the concentration of the various components present in the sample. Components with higher concentrations tend to produce elevated intensity values. In contrast, those with lower concentrations typically result in diminished intensity values. Oligopeptides are short-chain peptides composed of 2–20 amino acid residues (Apostolopoulos et al., 2021), and they are generally characterized by their high biological activity (Liu et al., 2024). We sorted the “PSM*intensity” values of the peptide sequences in descending order. We selected the top ten peptides, each containing fewer than 20 amino acids, for further analysis.

### 2.12 Network pharmacology analysis

#### 2.12.1 Action targets the prediction of bioactive peptides

Utilizing ChemDraw software 14.0.0.117, we constructed three-dimensional structures for the ten bioactive peptides based on their sequences. Subsequently, we employed Chem3D to perform energy minimization on these structures. Subsequently, upload the sequences of each bioactive peptide in “SMILE” format to Super-PRED (Gallo et al., 2022; Nagy et al., 2023) (https://prediction.charite.de/index.php) and SEA (Keiser et al., 2007) (https://sea.bkslab.org/) to obtain predicted targets for the 10 peptides. The sequences and mass spectra of the 10 peptide structures are presented in Table 2.

**TABLE 2:** Peptides of DHG identified by DHG proteomics database.

No.	Sequence	PSM	Intensity	Length	M (Da)
1	GFSGLDGAK (P1)	37	4.84 × 10^10^	9	850.4185
2	GFP(+15.99)GADGVAGPK (P2)	58	2.78 × 10^10^	12	1087.5298
3	GSAGPP(+15.99)GATGFP(+15.99)GAAGR (P3)	48	2.96 × 10^10^	17	1458.6851
4	GPP(+15.99)GPQGAR (P4)	29	4.61 × 10^10^	9	851.4249
5	GSP(+15.99)GEAGRP (+15.99)GEAGLP (+15.99)GAK (P5)	32	3.85 × 10^10^	18	1654.791
6	GVVGLP (+15.99)GQR (P6)	51	2.32 × 10^10^	9	897.5032
7	GPAGPQGPR (P7)	29	3.76 × 10^10^	9	835.43
8	GIP(+15.99)GEFGLP (+15.99)GPAGAR (P8)	55	1.88 × 10^10^	15	1426.7205
9	GPSGPQGIR (P9)	30	2.73 × 10^10^	9	867.4562
10	GLP (+15.99)GTAGLP (+15.99)GMK(+178.05)GHR (P10)	24	3.39 × 10^10^	15	1657.8093

#### 2.12.2 Collection of OP-related targets and acquisition of potential anti-OP targets of bioactive peptides

Simultaneously, obtain OP-related targets from Gene Cards (https://www.genecards.org/), DisGeNET (https://www.disgenet.org/), TTD (https://db.idrblab.net/ttd/), OMIM (https://omim.org/), PharmGKB (https://www.pharmgkb.org/), and DrugBank (https://www.drugbank.com/). Identify all targets among the 10 peptides, then use Venny 2.1 (https://bioinfogp.cnb.csic.es/tools/venny/index.html) to map the DHG bioactive peptide targets with the disease targets. The intersecting targets will be considered potential anti-OP targets for the bioactive peptides.

#### 2.12.3 Construction and topology analysis of PPI network of potential anti-OP targets

Import the potential anti-OP targets of the peptides into the STRING database (https://www.string-db.org/), setting the species to “*Homo Sapiens*” and hiding isolated nodes. Construct a protein-protein interaction (PPI) network with “moderate confidence” (0.400) and visualize it using Cytoscape (version 3.7.2, the National Institute of General Medical Sciences, Bethesda, MD, United States). The anti-OP core targets were selected based on degree, betweenness centrality (BC), and closeness centrality (CC).

#### 2.12.4 Pathway enrichment analysis

Import the potential anti-OP core targets of the peptides into the DAVID database for GO functional and KEGG pathway enrichment analysis. Use a threshold of *P* < 0.05 and visualize the top 20 GO terms and top 20 KEGG pathways using R 4.3.3.

#### 2.12.5 Molecular docking analysis of DHG bioactive peptide with potential key anti-OP targets

Download the molecular structure of potential key anti-OP targets of DHG active peptides from the RCSB PDB database (https://www.rcsb.org/). Before docking, use PyMOL 2.4 to remove the original ligands and water molecules and add hydrogen atoms. Set this as the receptor structure. Use Autodock Vina 1.2.0 to dock DHG bioactive peptides to the receptor. Evaluate binding affinity by comparing the “CDOCKER_Energy” values. Visualize the optimal conformation of DHG active peptides with potential anti-OP targets using PyMOL.

#### 2.12.6 Verification of the binding model using molecular dynamics simulations (MDS)

MDS was conducted using GROMACS 2020.6 (http://www.gromacs.org) software to evaluate the stability of the docking model (Jiang et al., 2025). The protein was modeled using the CHARMM36 force field, and small molecules were modeled using the GAFF force field. The system was solvated with TIP3P water molecules and neutralized with NaCl counterions to ensure charge balance. Before the simulation, the system was energy-minimized using the steepest descent and conjugate gradient methods. The system was equilibrated by running 100 ps NVT and NPT simulations separately to determine the temperature and pressure. Each system was simulated for 100 ns at a temperature of 310 K and a pressure of 1.0 bar.

### 2.13 Effect of DHG active peptide P2 on bone formation

#### 2.13.1 Mineralization assay

Rat bone marrow mesenchymal stem cells (BMSCs) were maintained in α-MEM supplemented with 10% FBS and 1% penicillin/streptomycin. Cell cultures were incubated at 37 °C in a humidified atmosphere containing 5% CO_2_. BMSCs were seeded in 96-well plates at a density of 10,000 cells/well. After adhering for 24 h, replace the osteogenic differentiation medium containing LY 294002 (10 μM) and P2 (20 μg/mL) according to the grouping, with four wells per group. Following 14-day induction, cells were washed twice with PBS and stained with ARS solution for 20 min at room temperature. Quantification of mineralized nodules was performed using high-power field microscopy (Nikon Eclipse Ti2; ×200 magnification). The mineralized area was quantified using ImageJ 1.52v software (National Institutes of Health, MD, United States) with standardized threshold settings (RGB threshold range: 120–255).

#### 2.13.2 Western blotting

Culture BMSC cells at 10^6^ cells/well in six-well plates, with three replicates per group. After 24 h, replace the medium with an osteogenic differentiation medium containing LY 294002 (10 μM) and P2 (20 μg/mL) according to the group allocation. After incubation for 24 h, use RIPA buffer supplemented with protease/phosphatase inhibitors to extract proteins and detect the expression of key PI3K/Akt pathway proteins, including p-AKT, AKT, p-ERK, and ERK. After 7 days of incubation, extract the proteins to detect the expression of RUNX2 protein. Extract the proteins and determine the concentration according to the manufacturer’s instructions using a BCA protein assay kit. Use 10% SDS-PAGE to separate the proteins (10 μg per lane), and then transfer the gel to a PVDF membrane using wet transfer at 350 mA for 70 min. Block the membrane in TBST with 5% BSA (10 mM Tris-HCl, 150 mM NaCl, 0.1% Tween 20) at 4 °C overnight. Incubate the membrane with primary antibodies at 4 °C overnight, then wash with TBST. Next, incubate with anti-rabbit secondary antibodies at room temperature for 1 h. After washing with TBST, visualize the immunoreactive signals using Luminol reagents. ImageJ was used to analyze its grayscale value.

### 2.14 Analysis of common targets between cardiovascular diseases (CVDs) and OP

To investigate the multi-organ associations of chemotherapy-induced side effects, a network pharmacology analysis was performed to elucidate the shared molecular mechanisms between CVDs and OP. First, targets associated with “cardiovascular diseases” were retrieved from the GeneCards database. Subsequently, the Venny 2.1 online tool was utilized to conduct an intersection analysis between the CVDs-related targets and the OP-related targets (as identified in Section 2.12.2), thereby identifying the common targets. These common targets were then imported into the STRING database to construct a PPI network (species limited to *Homo Sapiens*, confidence >0.400, hide isolated nodes). The topological parameters of the resulting PPI network were analyzed using Cytoscape software. Targets with values for degree, BC, and CC that were all greater than their respective median values were designated as core targets for the CVDs-OP interaction. Finally, KEGG pathway enrichment analysis was conducted on these core targets using the DAVID database. Pathways meeting the significance threshold of *P* < 0.05 were identified, and the top 20 enriched pathways were selected for further analysis.

### 2.15 Statistical analysis

Statistical analyses of the collected data were performed using GraphPad Prism 7.0 or Microsoft Excel 2007, as appropriate for the dataset. The results of these analyses have been reported as the mean ± standard error of the mean ( $\bar{x}$ ± SEM). For comparisons across multiple experimental groups, one-way analysis of variance (ANOVA) was employed, incorporating Dunnett’s adjustment for multiple comparisons. When assessing the rates between two distinct groups, a chi-square test was utilized. *P* < 0.05 was predetermined to be the threshold for statistical significance.

## 3 Results

### 3.1 DHG increases BMD of CTX-induced OP rats

**FIGURE 1:**
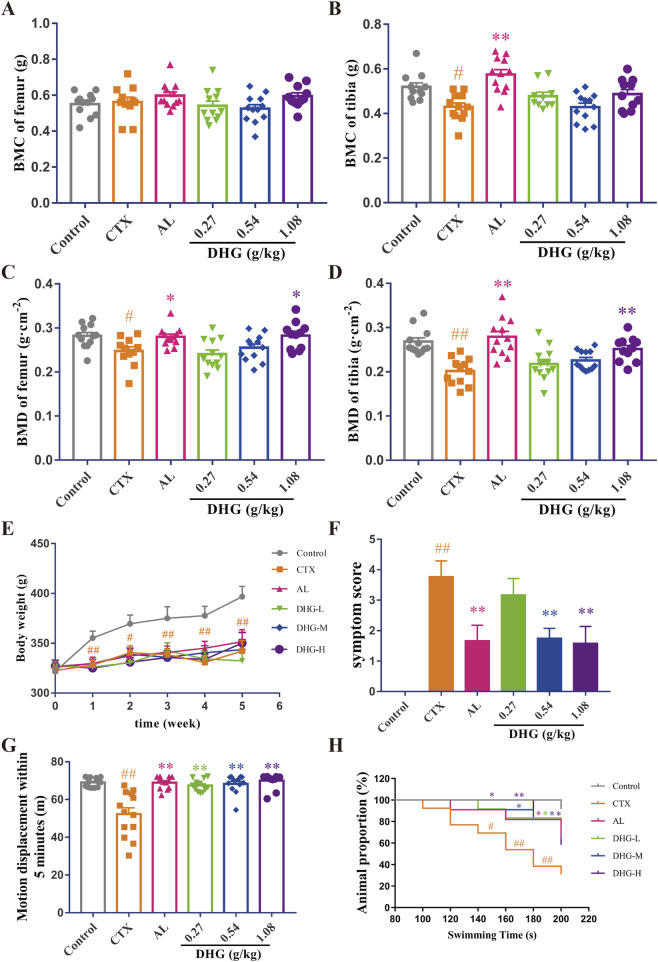
Improving effect of DHG on bone and physical signs of CTX-induced OP rats. **(A)** Femoral and **(B)** tibial BMC. **(C)** Femoral and **(D)** tibial BMD. **(E)** Changes in weekly body mass. **(F)** Changes in fifth week symptom scores. **(G)** Motor displacement in 5 min. **(H)** Proportion of animals in different swimming periods. Values are expressed as mean ± standard error of the mean (*n* = 11–12 rats per group). Statistical significance was evaluated using one-way ANOVA. Multiple comparisons between groups were performed using the Dunnett test, and the Chi-square test was applied to compare the inter-group rates. ^#^ *P* < 0.05, ^##^ *P* < 0.01 vs. Control in the same week; ^*^ *P* < 0.05, ^**^ *P* < 0.01 vs. CTX in the same week. Abbreviations: CTX, cyclophosphamide; AL, alendronate (7.35 mg/kg); DHG-L, deer-hide gelatin low dose (0.27 g/kg); DHG-M, deer-hide gelatin medium dose (0.54 g/kg); DHG-H, deer-hide gelatin high dose (1.08 g/kg).

The BMC and BMD are vital indicators used for clinical diagnosis and monitoring of osteoporosis (Ward et al., 2019; Löffler et al., 2021). To further explore whether DHG promotes bone architecture integrity in rats, dual-energy X-ray absorptiometry was used to evaluate the BMC and BMD of the femur and tibia in trial rats. The difference in femoral BMC was not significant among all groups (*P* > 0.05), as seen in Figure 1A. As shown in Figures 1B–D, the CTX group displayed significant reductions in tibial BMC, femoral BMD, and tibial BMD, confirming that the CTX-induced osteoporotic model was successfully established. The DHG at 1.08 g/kg markedly reversed the declines in femoral (*P* < 0.05) and tibial (*P* < 0.01) BMD induced by CTX, as shown in Figures 1C,D. These findings indicated that DHG had a therapeutic effect in mitigating CTX-induced bone damage by increasing BMD in the OP rats.

### 3.2 Improving the effect of DHG on physical signs of CTX-induced OP rats

To investigate the effects of DHG from the angle of physical signs, different doses were orally administered to nine-week-old male CTX-induced OP rats for 5 weeks. As depicted in Figure 1E, all body weights decreased significantly after CTX modeling (*P* < 0.05). Although no statistical difference was observed, DHG treatment still showed a trend toward increased body weight. The symptoms score was measured to assess the overall status of CTX damage. The increased symptoms score in the CTX group was effectively ameliorated by 0.54 and 1.08 g/kg DHG (*P* < 0.01), as seen in Figure 1F and Supplementarty Figure S1. To assess the effects of DHG on locomotor activity and physical strength quantitatively, motor displacement was measured over 5 min. Rats modeled with CTX exhibited a decline in displacement, indicating a loss of locomotor activity and physical strength. DHG treatment ameliorated this CTX-induced fatigue (*P* < 0.01, Figure 1G). To evaluate the endurance of the rats, a weight-bearing swimming test was conducted to monitor their performance. As shown in Figure 1H, CTX-induced fatigue rats displayed markedly decreased perseverance proportion within the time frame of 140–200 s (*P* < 0.05). In contrast, rats in the DHG-L, DHG-M, and DHG-H groups showed a marked increase in endurance proportion across a broader time range, including 140–200 s, 180–200 s, and 140–200 s (*P* < 0.05), respectively. The cumulative data suggested that DHG enhanced the swimming endurance of rats subject to CTX treatment. These overall improvements in the physical ability of OP model rats supported the potential of DHG in ameliorating CTX-induced functional impairments.

### 3.3 DHG enhances tibial biomechanical strength in CTX-induced OP rats

**FIGURE 2:**
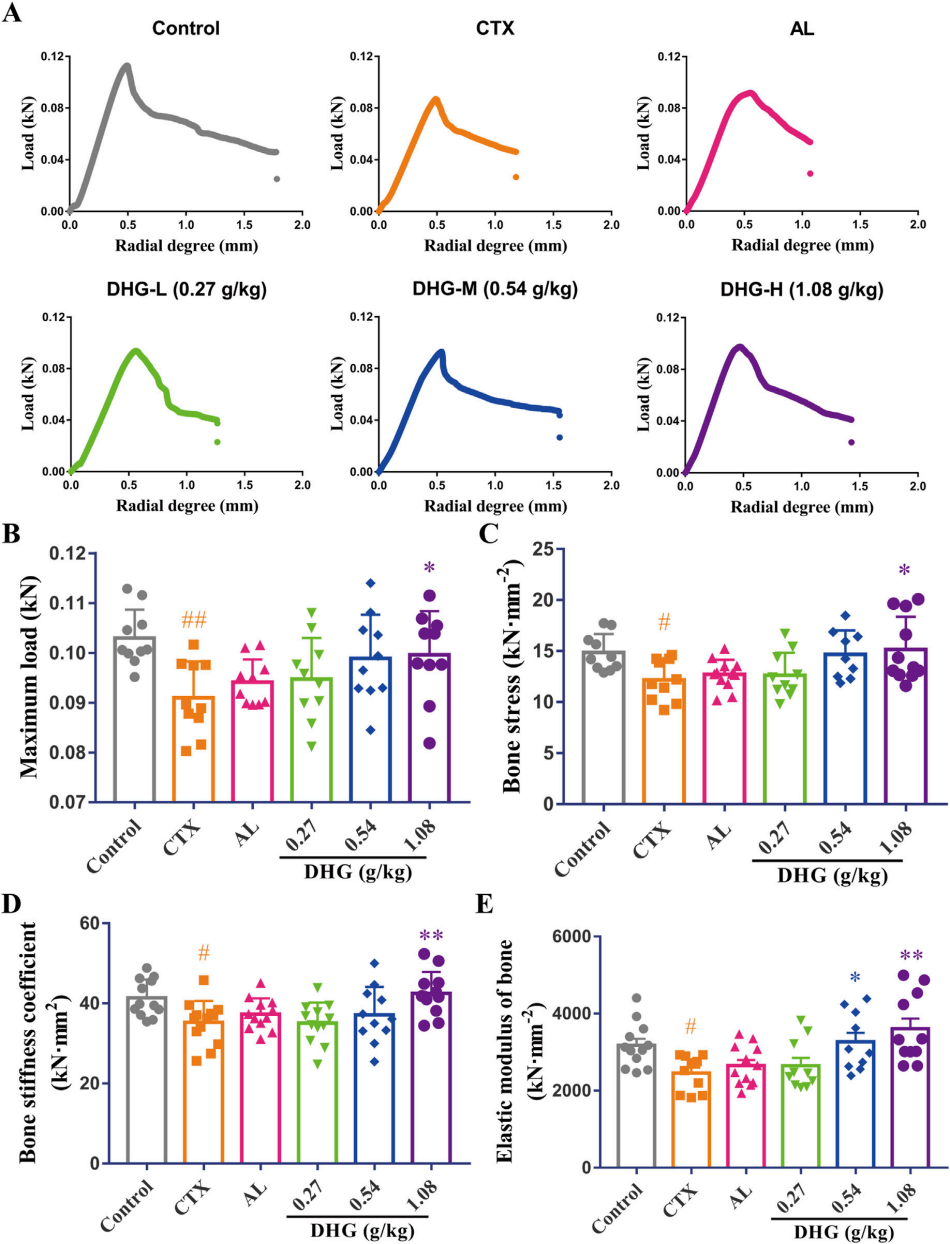
DHG enhances tibial biomechanical strength in OP rats. **(A)** Radial degree-load relationship diagram. **(B)** Maximum load. **(C)** Bone stress, **(D)** stiffness coefficient, and **(E)** elastic modulus. Values are expressed as mean ± standard error of the mean (*n* = 9–12 rats per group). Statistical significance was evaluated by one-way ANOVA. ^#^ *P* < 0.05, ^##^ *P* < 0.01 vs. Control; ^*^ *P* < 0.05, ^**^ *P* < 0.01 vs. CTX. Abbreviations: CTX, cyclophosphamide; AL, alendronate (7.35 mg/kg); DHG-L, deer-hide gelatin low dose (0.27 g/kg); DHG-M, deer-hide gelatin medium dose (0.54 g/kg); DHG-H, deer-hide gelatin high dose (1.08 g/kg).

**FIGURE 3:**
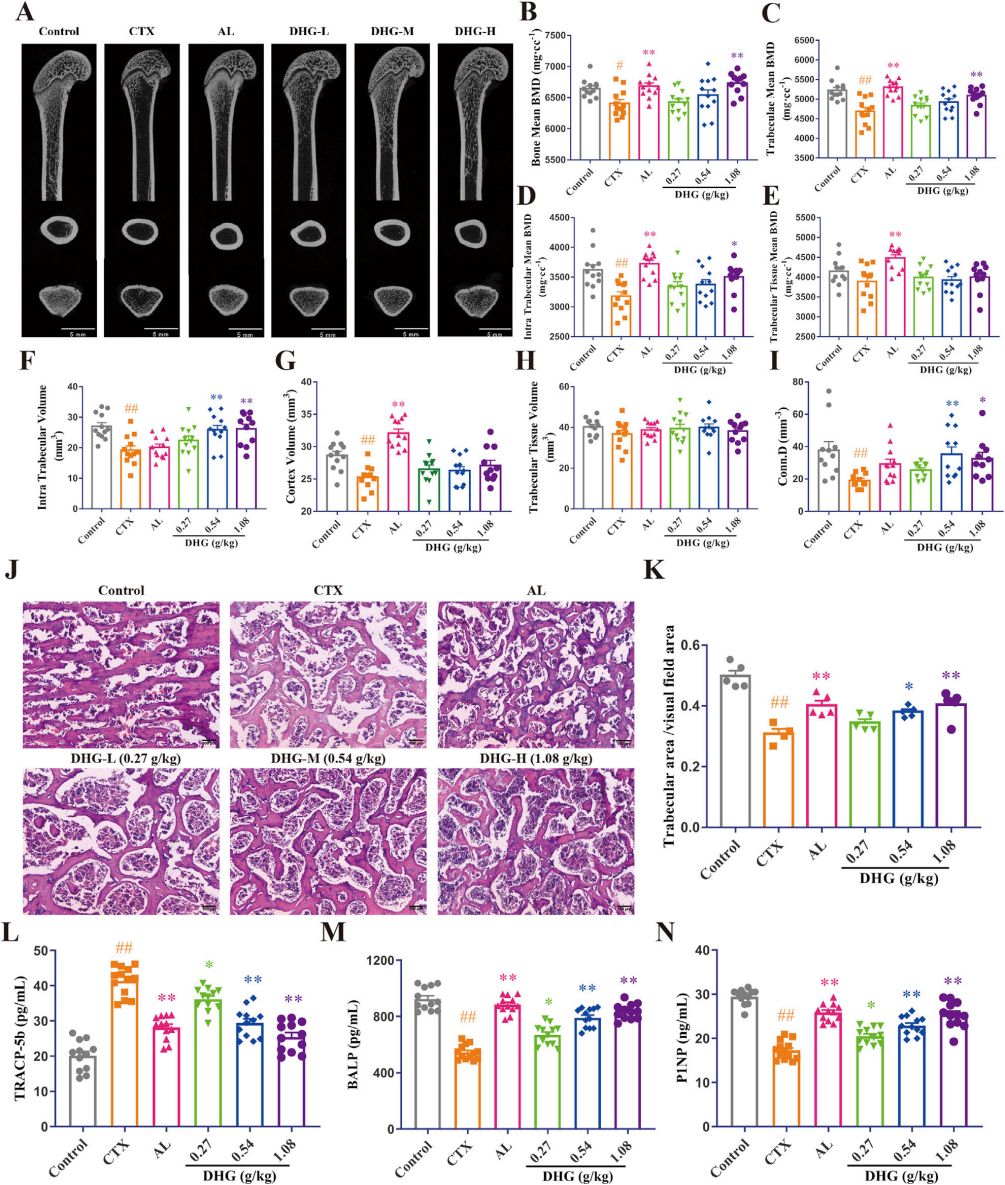
DHG improves the trabecular microstructure of CTX-induced OP rats. **(A)** CT images of the femur. Mean **(B)** bone, **(C)** trabeculae, **(D)** intra trabecular, **(E)** trabecular tissue BMD, **(F)** intra trabecular, **(G)** cortex, **(H)** trabecular tissue volume, and **(I)** connectivity density (*n* = 11-12 rats per group). **(J)** Structural changes of tibial trabeculae by HE staining (magnification: ×100, scale bar, 10 μm). **(K)** Trabecular area statistics (*n* = 4-5 rats per group). Expression of **(L)** TRACP-5b, **(M)** BALP, and **(N)** P1NP was detected in serum by ELISA (*n* = 11–12 rats per group). Values are expressed as mean ± standard error of the mean. Statistical significance was evaluated by one-way ANOVA. ^#^ *P* < 0.05, ^##^ *P* < 0.01 vs. Control; ^*^ *P* < 0.05, ^**^ *P* < 0.01 vs. CTX. Abbreviations: CTX, cyclophosphamide; AL, alendronate (7.35 mg/kg); DHG-L, deer-hide gelatin low dose (0.27 g/kg); DHG-M, deer-hide gelatin medium dose (0.54 g/kg); DHG-H, deer-hide gelatin high dose (1.08 g/kg); TRACP-5b: tartrate-resistant acid phosphatase 5b; BALP: bone-specific alkaline phosphatase; P1NP: N-terminal propeptide of type I procollagen.

Bone fractures and fragility in OP directly reflect the reduced biomechanical changes resulting from diminished bone structure (Fields and Keaveny, 2012; Ibrahim et al., 2020). A three-point bending test was used to investigate the effect of DHG on bone biomechanics. As shown in Figure 2A, the radial degree-load relationship diagram of the CTX group was significantly changed compared with the control group. After DHG treatment, the variation in the curve decreased and became more similar to that of the control group. Further statistics on these data are shown in Figures 2B–E. Maximum load and bone stress reflect the load-bearing capacity of the entire tibia and its per unit area, respectively. The bone stiffness coefficient indicates the strength of the bone. The above parameters showed noticeable declines after CTX treatment (*P* < 0.01, *P* < 0.05, and *P* < 0.05, respectively). The impact of CTX was significantly improved after DHG (1.08 g/kg) treatment (*P* < 0.05, *P* < 0.05, *P* < 0.01, respectively), indicating DHG effectively improves bone hardness and strength, as seen in Figures 2B–D. Elastic modulus reflects the elasticity of bone. The CTX-induced OP group showed a marked decrease in elastic modulus. The DHG treatment at 0.54 and 1.08 g/kg increased elastic modulus in Figure 3E, suggesting that DHG enhanced both the elasticity and toughness of bone. Collectively, these enhancements in biomechanical attributes indicate that DHG has a bone-strengthening effect following CTX-induced OP.

### 3.4 DHG improves the trabecular microstructure of CTX-caused OP rats

To further evaluate the therapeutic effects of DHG and its impact on changes in bone microstructure, a micro-CT scan was conducted to assess femoral bone integrity quantitatively. As shown in Figure 3A, the trabecular density and structure of the femur in the CTX group were significantly damaged compared with the control group, and after DHG treatment, the variation was improved. Further statistics on these data are shown in Figures 3B–I. The results of BMD showed that high-dose DHG markedly increased femoral BMD (*P* < 0.01), as seen in Figure 3B, consistent with the X-ray results. Trabecular or cancellous bone plays a crucial role in the overall mechanical properties and health of the skeletal system (Fields and Keaveny, 2012). The DHG treatment increased trabeculae mean BMD (*P* < 0.05) and intra-trabecular BMD (*P* < 0.01), as seen in Figures 3C–E, but no significant changes were observed in trabecular tissue BMD. In addition, both intra-trabecular volume and connectivity density were significantly increased in a dose-dependent manner after DHG treatment compared to the CTX group. At the same time, no significant changes were observed in cortex volume and trabecular tissue volume, as seen in Figures 3F–I. Collectively, these microstructural parameter changes highlight the potential use of DHG for promoting bone formation and protection.

To further observe the changes in the trabecular bone at the tissue level in detail, HE staining was performed simultaneously on the tibia. Histopathologic analysis by HE staining revealed a marked degradation of the trabecular architecture in the CTX group. In contrast to the Control group (Figure 3J), which exhibited uniformly thick trabeculae with proper interconnections, the CTX group (Figure 3K) demonstrated severe trabecular fracture, extensive disconnection at trabecular junctions, increased marrow/void space, and a reduced trabecular number, resulting in a significant decrease in trabecular bone area (*P* < 0.01). The trabecular impairments were significantly ameliorated by DHG in a dose-dependent manner, a result consistent with the micro-CT findings. Taken together, these data indicate that DHG promotes bone health and exhibits protective properties in osteoporotic conditions.

### 3.5 DHG regulates bone remodeling marker TRACP-5b, BALP, and P1NP

**FIGURE 4:**
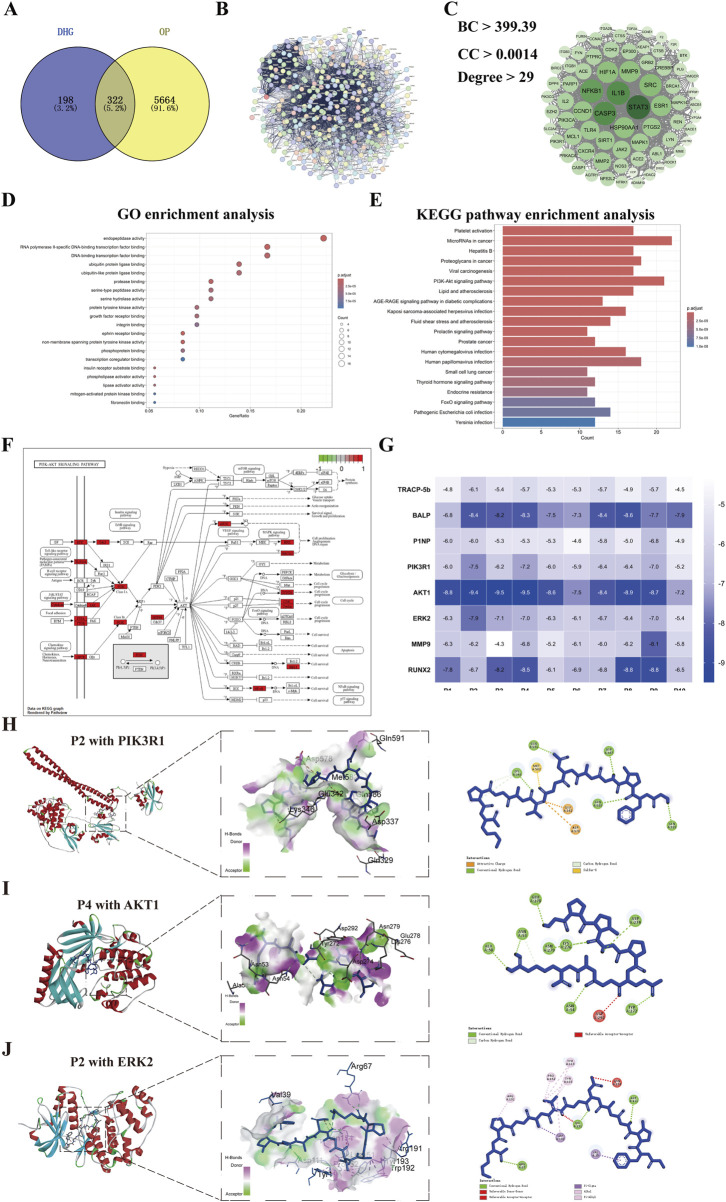
Network pharmacology analysis of DHG treating OP. **(A)** Distribution of DHG and OP target genes, **(B)** Protein-protein interactions network of 322 key targets of DHG treating OP, **(C)** Protein-protein interactions network of 76 core targets of DHG treating OP, **(D)** Top 20 results for GO enrichment analysis, **(E)** Top 20 pathways for KEGG enrichment analysis. **(F)** PI3K signaling pathway map from KEGG database, **(G)** Heatmap of binding energies (kcal/mol) for molecular docking results of the DHG active peptides with the key targets. Molecular docking results of DHG active peptides **(H)** P2 with PIK3R1, **(I)** P4 with AKT1, **(J)** P2 with ERK2 (MAPK1). PI3K: phosphatidylinositol 3 kinase. PIK3R1: phosphoinositide-3-kinase regulatory subunit 1; AKT1: v-akt murine thymoma viral oncogene homolog 1; ERK2: extracellular regulated protein kinases 2.

To assess the effect of DHG on repairing CTX-induced bone metabolic disruption, ELISA was used to determine bone metabolism-related biochemical indicators in serum. A marker of bone resorption, TRACP-5b, increased in the CTX-treated group. It then markedly decreased after DHG treatment compared to the CTX group, as seen in Figure 3L. Anabolic indicators of bone formation activity, including BALP and P1NP (Schini et al., 2022), were significantly reduced in the CTX group. The DHG-L, DHG-M, and DHG-H treatments reduced the CTX-induced reduction in BALP and P1NP, as seen in Figures 3M,N, implying an improvement in osteoblastic function and bone formation. Additionally, the molecular docking results further demonstrated that DHG active peptides exhibit strong binding interactions with these biomarkers, as illustrated in Supplementarty Figures S2A–S2C (the figure illustrates the optimal docking results for the ten peptides under investigation) and Figure 4G. These results suggest that DHG may potentially rectify the CTX-induced damage to bone microstructure by reducing osteoclastogenic activity and supporting bone formation processes.

### 3.6 Network pharmacology results of DHG bioactive peptide treating OP

A total of 520 common action targets of 10 DHG bioactive peptides were obtained after the prediction of Super-PRED and SEA databases, and 5986 OP-related targets were provided by DisGeNET, GeneCards, TTD, PharmGKB, OMIM, and DrugBank databases. Then, a total of 322 intersection targets were selected as the potential anti-OP targets of DHG bioactive peptide by matching the action targets of DHG bioactive peptide with the OP-related targets (Figure 4A). The 322 target proteins were imported into the STRING database to establish a PPI network, as illustrated in Figure 4B. Subsequently, employing BC, DC, and CC metrics, a selection process yielded 72 core targets, with their corresponding PPI network relationships depicted in Figure 4C. The core target PPI network reveals that the seven targets with the highest degree values are STAT3, CASP3, NFKB1, IL1B, HSP90AA1, HIF1A, and MMP9, suggesting that DHG active peptide may exhibit the anti-OP efficacy mediated by interactions between multiple targets. These 72 targets were imported into STRING to establish a PPI network (Figure 4C), suggesting that the DHG active peptide may exhibit anti-OP efficacy mediated by interactions between multiple targets. To further elucidate the anti-OP mechanism of DHG bioactive peptides, the above 72 targets were conducted GO function and KEGG pathway enrichment analysis using the DAVID database. A total of 122 terms were obtained after GO function enrichment analysis (*P* < 0.05). The top 20 significantly enriched GO functions are displayed in Figure 4D. The GO function analysis results suggest that DHG bioactive peptides anti-OP targets may participate in molecular functions, such as endopeptidase activity, protease binding, protein tyrosine kinase activity, growth factor receptor binding, integrin binding, and ephrin receptor binding.

A total of 159 KEGG pathways were enriched (*P* < 0.05). The top 20 significantly enriched KEGG pathways are displayed in Figure 4E. The results revealed that the potential anti-OP targets of the DHG active peptide play important roles in bone reconstruction, mainly by participating in the microRNA in cancer, PI3K-AKT signaling pathway, and Thyroid hormone signaling pathway. Among them, 21 targets participated in the PI3K/AKT signaling pathway (Figure 4F), e.g., PIK3R1, PIK3CA, HSP90AA1, ERK2 (MAPK1), NFKB1. In addition, it was found that a target simultaneously participates in multiple pathways, e.g., PIK3R1, PIK3CA, ERK2 (MAPK1), and NFKB1. Further docking studies were conducted on the active peptides of PIK3R1, AKT, ERK2, and DHG, with the docking scores presented in Figure 4G. The docking results for the DHG active peptides are illustrated in Figures 4H–J (the figure illustrates the optimal docking results for the ten peptides under investigation) demonstrate favorable binding interactions with PIK3R1, AKT1, and ERK2.

**FIGURE 5:**
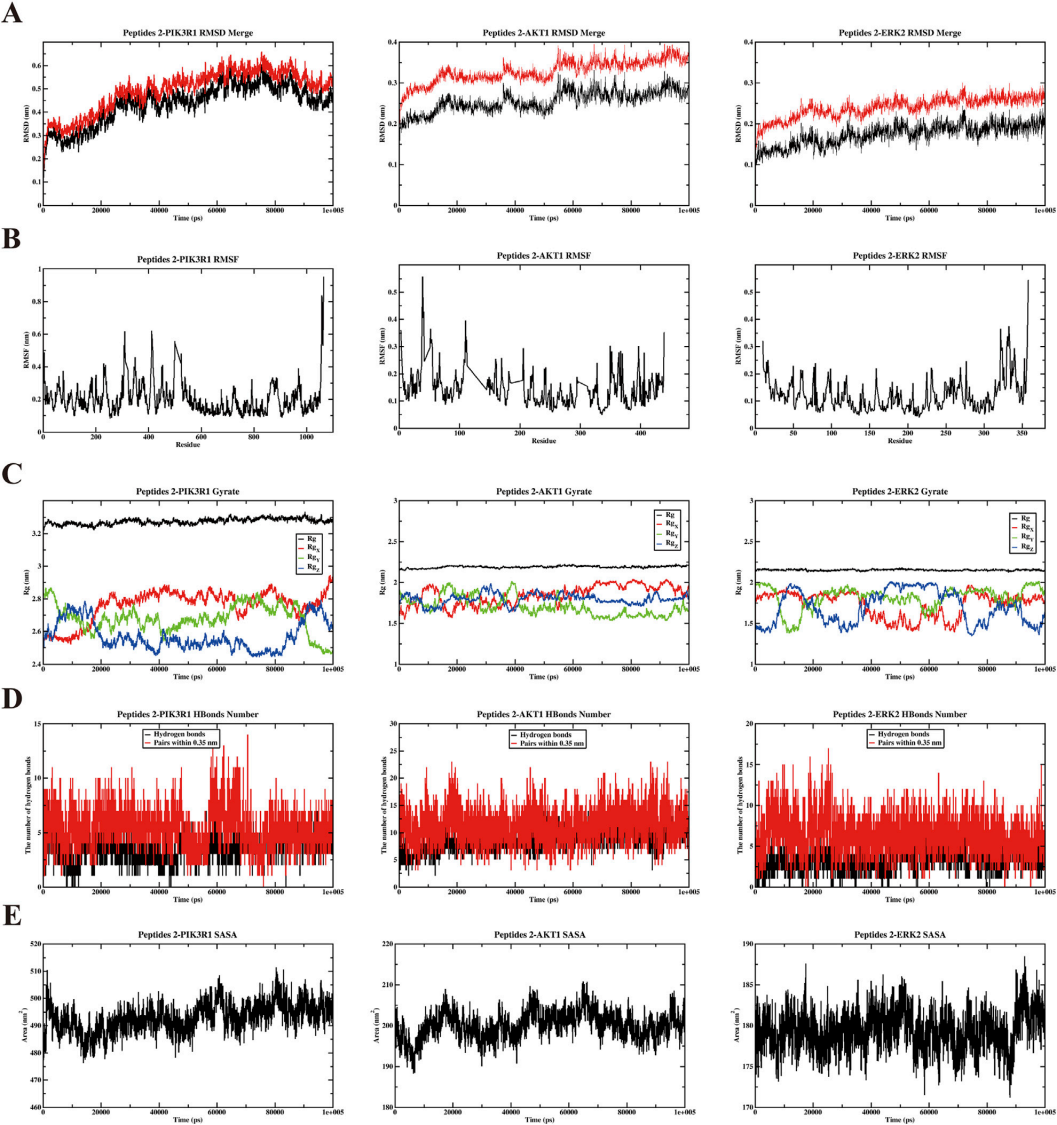
Results of molecular dynamics simulation (MDS) analysis illustrating. **(A)** Root mean square deviation (RMSD), **(B)** root mean square fluctuation (RMSF), **(C)** radius of gyration (Rg), **(D)** the number of H bonds, and **(E)** solvent accessible surface area (SASA) for three distinct proteins and P2 complexes: PIK3R1, AKT1, ERK2.

To further evaluate the stability and dynamic interactions of the DHG active peptide binding with PIK3R1, AKT1, and ERK2 proteins, based on molecular docking results, a representative active peptide, P2, was selected for molecular dynamics simulations. Key parameters were analyzed, including root mean square deviation (RMSD), root mean square fluctuation (RMSF), radius of gyration (Rg), hydrogen bonding, and solvent accessible surface area (SASA). The RMSD values of the protein complexes ranged from 0.1 to 0.6 nm (Figure 5A), indicating good dynamic stability during the 100-ns simulation period. Figure 5B shows that all amino acid residues in the three P2 protein complexes have fluctuations less than 1 nm, further confirming the stability of the complexes. Analysis of the Rg provides insights into the structural folding and compactness of molecules during molecular dynamics simulations. The results indicate that the Rg values of the three complexes are consistently smaller than that of the individual protein, suggesting the formation of a stable structure (Figure 5C). Furthermore, analysis of the main-chain hydrogen bonding indicates the presence of stable hydrogen bonds in the complex (Figure 5D). Analysis of SASA helps to understand the compactness of proteins. The results showed that the SASA curves of the three P2 protein complexes remained relatively stable throughout the simulation process (Figure 5E). Collectively, these results suggested that the anti-OP effects of DHG active peptides may be associated with the modulation of the PI3K/AKT signaling pathway.

### 3.7 DHG inhibits MMP9 and promotes RUNX2 protein expression

**FIGURE 6:**
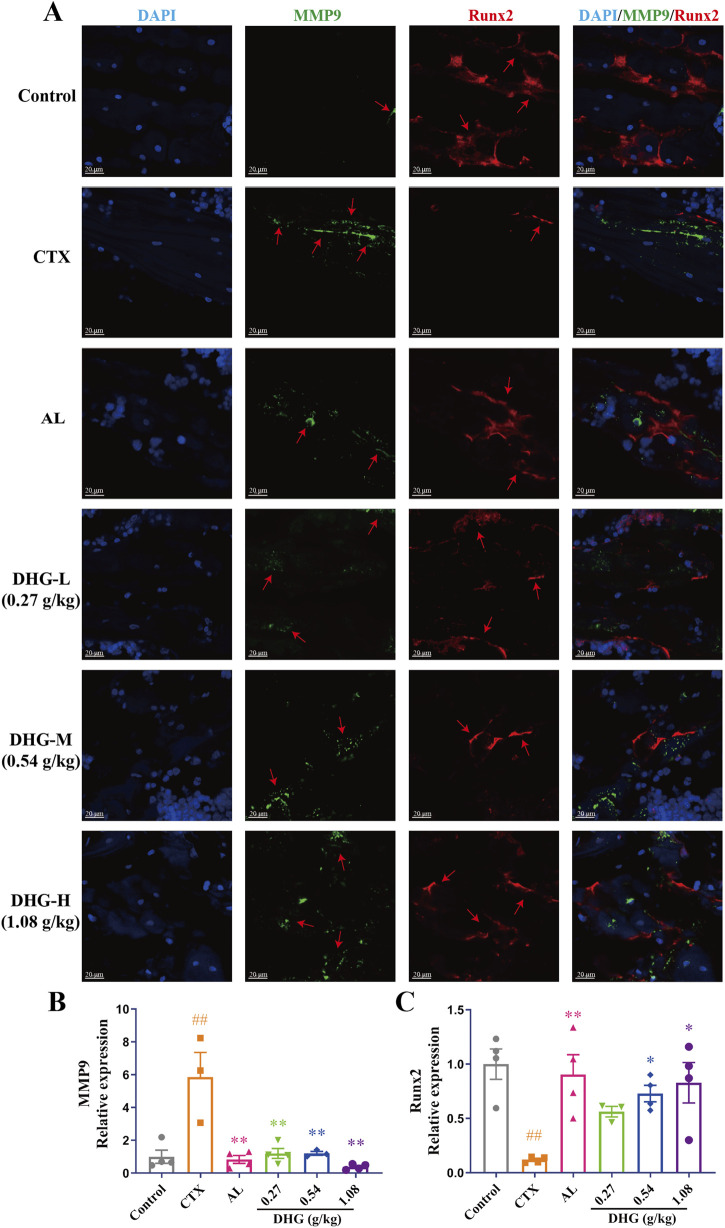
DHG inhibits MMP9 and promotes Runx2 protein expression. **(A)** Immunofluorescence staining of tibial sections. Blue: DAPI; green: MMP9; red: Runx2; magnification: ×200, scale bar: 20 μm. Statistics of **(B)** MMP9 and **(C)** Runx2 expression in the tibia. Values are expressed as mean ± standard error of the mean (*n* = 3–4 rats in each group). Statistical significance was evaluated by one-way ANOVA. ^##^ *P* < 0.01 vs. Control; ^*^ *P* < 0.05, ^**^ *P* < 0.01 vs. CTX. Abbreviations: CTX, cyclophosphamide; AL, alendronate (7.35 mg/kg); DHG-L, deer-hide gelatin low dose (0.27 g/kg); DHG-M, deer-hide gelatin medium dose (0.54 g/kg); DHG-H, deer-hide gelatin high dose (1.08 g/kg); MMP9: matrix metalloproteinase-9; Runx2: runt-related transcription factor 2.

The MMP9 promotes osteoclastogenic activation and bone resorption (Zhu et al., 2020a). Moreover, the findings from the PPI network indicated that MMP9 functions as a pivotal target for the anti-OP properties of DHG (Figure 4C). RUNX2 is a key transcription factor in bone formation, regulating osteoblast differentiation and bone generation (Zhu et al., 2024). A groundbreaking study demonstrates that RUNX2 and the PI3K/AKT pathway are interdependent in regulating the differentiation and migration of both osteoblasts and chondrocytes (Fujita et al., 2004). Immunofluorescence assessment provided further insight into the molecular mechanisms of DHG treatment. The expression of MMP9 was significantly elevated in the CTX group (*P* < 0.01), as seen in Figures 6A,B. In contrast, all DHG groups showed a significant decrease in MMP9 expression (*P* < 0.01), indicating that DHG effectively inhibited bone resorption. As shown in Figures 6A,C, RUNX2 expression significantly decreased in the CTX-induced OP rats (*P* < 0.01). The use of DHG at 1.08 g/kg reversed this decline in RUNX2 expression, suggesting that DHG restored osteoblast activity and promoted bone formation in CTX-induced bone degradation. Moreover, the molecular docking results further confirmed that DHG active peptides exhibit strong binding interactions with MMP9 and RUNX2, as shown in Supplementary Figures S2D, S2E (the figure illustrates the optimal docking results for the ten peptides under investigation) and Figure 4G. This suggested that these interactions may play a significant role in the biological activities of DHG peptides, particularly in bone metabolism and remodeling. These results preliminarily demonstrated the molecular mechanism by which DHG inhibits bone resorption and promotes bone formation.

### 3.8 DHG active peptide promotes bone formation through the PI3K/AKT pathway

**FIGURE 7:**
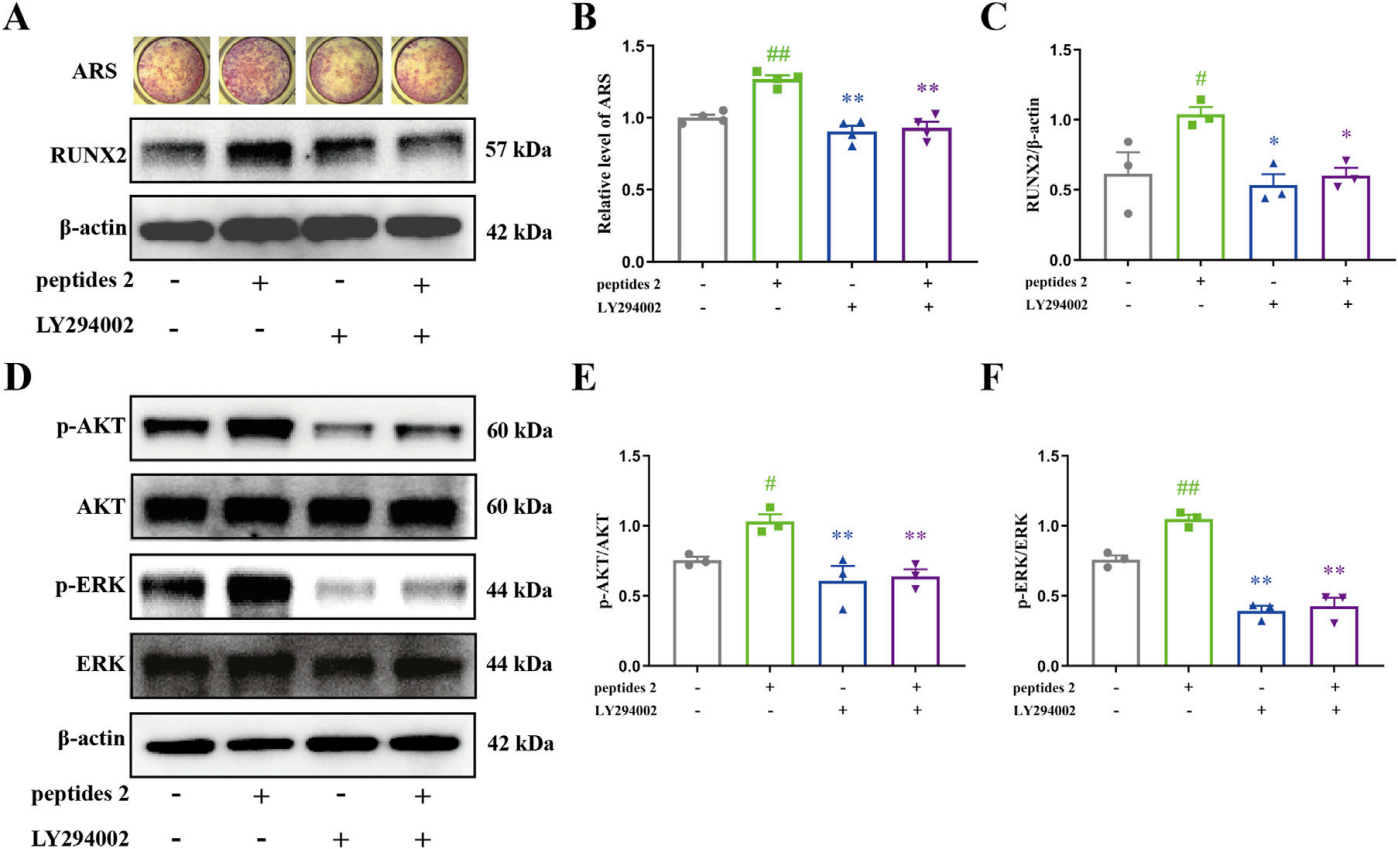
Effect of DHG active peptide P2 on the bone formation of BMSCs. **(A)** Effect of P2 and LY294002 on mineralization and expression levels of RUNX2 proteins, **(B)** Mineralization area analysis (*n* = 4 in each group), **(C)** analysis of expression levels of RUNX2 proteins (*n* = 3 in each group). **(D)** Effect of P2 and LY294002 on the expression levels of p-AKT, AKT, p-ERK, and ERK proteins. Analysis of expression levels of **(E)** p-AKT and **(F)** p-ERK proteins (*n* = 3 in each group). Data are expressed as the means ± standard error of the mean. Statistical significance was evaluated by one-way ANOVA. ^#^ *P* < 0.05, ^##^ *P* < 0.01 vs. Control; ^*^ *P* < 0.05, ^**^ *P* < 0.01 vs. peptides 2. AKT1: v-akt murine thymoma viral oncogene homolog 1; p-AKT: phosphorylation v-akt murine thymoma viral oncogene homolog 1; ERK2: extracellular regulated protein kinases 2; p-ERK2: phosphorylation extracellular regulated protein kinases 2; Runx2: runt-related Transcription Factor 2.

To further investigate the anti-OP effects and mechanisms of DHG, the impact of the DHG active peptide P2 on bone formation was studied. Mineralization is the final step in osteoblast differentiation and serves as a direct indicator of the extent of bone formation. The intense red complexation reaction between Ca^2+^ and ARS in mineralized nodules visually reflects this process (Wu et al., 2020b). To assess the effect of the DHG active peptide on bone mineralization, an ARS staining analysis was conducted to evaluate osteoblastic differentiation via the formation of mineralized nodules. As shown in Figure 7A, mineralization was induced after 7 days, and P2 promoted mineralization. Quantitative mineralization analysis (Figure 7B) indicated that P2 significantly enhanced the formation of mineralization nodules (*P* < 0.01), while LY294002 significantly inhibited the formation of P2-induced mineralization nodules (*P* < 0.01). Additionally, Western blot results (Figures 7A,C) showed that P2 significantly upregulated RUNX2 expression (*P* < 0.01), while LY294002 significantly inhibited the upregulation of RUNX2 induced by P2 (*P* < 0.01). These results suggested that the DHG active peptide P2 effectively promotes osteocyte differentiation, and this effect can be inhibited by the PI3K inhibitor LY294002.

The PI3K/AKT/ERK pathway represents a crucial mechanism that regulates cell survival and proliferation (Manning and Toker, 2017). To clarify how the DHG active peptide affects osteoblasts via this pathway, the expression levels of associated proteins were measured. As shown in Figures 7D–F, there was no significant difference in the expression of AKT and ERK; however, the active forms, p-AKT and p-ERK, were significantly upregulated with P2 intervention (*P* < 0.05 and *P* < 0.01, respectively). To further confirm whether the PI3K/AKT/ERK signaling pathway is involved in P2-induced osteogenesis, the combined effect of P2 and the PI3K inhibitor LY294002 was studied. LY294002 significantly inhibited the activation of AKT and ERK induced by P2 (Figures 7D–F, *P* < 0.01). This is consistent with the previous molecular docking results. In conclusion, the DHG active peptide P2 promotes bone formation by activating the PI3K/AKT/ERK signaling pathway in BMSCs, enhancing the phosphorylation of AKT and ERK, and upregulating the expression of RUNX2.

### 3.9 Results of the network pharmacology analysis of common targets between CVDs and OP

To investigate the shared pathophysiological mechanisms between CVDs and OP, a network pharmacology analysis was conducted. Through comprehensive database searches, we collected 8137 CVDs-related targets and 5,986 OP-related targets, respectively. Intersection analysis using the Venny 2.1 tool identified a total of 3,878 common targets (Supplemenatry Figure S3A), which may play a role in the pathogenesis and progression of both diseases. After constructing a PPI network with these common targets (Supplementary Figure S3B), a total of 683 core targets were screened through topological analysis (degree, BC, and CC all greater than the median). As shown in Supplementary Figure S3C, the top 15 targets, ranked by degree, are GAPDH, ACTB, AKT1, TP53, ALB, MYC, TNF, INS, CTNNB1, EGFR, IL-6, STAT3, JUN, IL-1β, and BCL2. KEGG pathway enrichment analysis of these core targets revealed that they were significantly enriched in pathways such as the PI3K-Akt signaling pathway, the MAPK signaling pathway, Pathways in cancer, Human cytomegalovirus infection, and Human T-cell leukemia virus 1 infection, among others (Supplementary Figure S3D). These results indicate that CVDs and OP share multiple key biological pathways at the molecular level, particularly the PI3K-AKT and MAPK pathways. This provides an essential theoretical basis for understanding the intrinsic link between the two diseases and for developing potential common therapeutic strategies.

## 4 Discussion

While effective against tumor cells, chemotherapeutic agents often have toxic effects on healthy tissues, leading to a range of adverse reactions, including fatigue, weakness, and weight loss (van den Boogaard et al., 2022). Consistent with this, our study observed a general decline in the health status of rats after CTX-induced OP, evidenced by weight loss and decreased physical strength. Encouragingly, DHG treatment significantly ameliorated these physical symptoms, indicating its ability to improve systemic health indicators affected by chemotherapy.

Normal bone metabolism relies on a dynamic balance between bone formation and bone resorption (McNeish et al., 2021; Niedźwiedzki and Filipowska, 2015). Chemotherapy-induced bone loss is typically attributed to decreased osteoblast-mediated bone formation (Huang et al., 2018) and enhanced osteoclast-mediated bone resorption (Pimenta-Lopes et al., 2023). The *in vivo* and *in vitro* experimental results of this study collectively form a complete chain of evidence elucidating the bone-protective mechanisms of DHG. In the animal model, DHG effectively reversed the CTX-induced decline in BMD, reduction in bone biomechanical strength, and destruction of trabecular bone microstructure. These macroscopic improvements are attributable to the systemic regulation of bone metabolism by DHG, as it significantly upregulated serum osteogenic markers BALP and P1NP (Takehana et al., 2018; Choi et al., 2019) while downregulating the osteoclastic marker TRACP-5b (Gossiel et al., 2022). Runx2 is known to regulate osteoblast differentiation, and studies suggest that Runx2 can regulate the expression of MMP9 (Pratap et al., 2005). Inhibition of MMP9-mediated citrate metabolism in BMSCs promotes the osteogenic differentiation of BMSCs (Da et al., 2024). Mechanistically, DHG promoted the expression of the key osteogenic transcription factor RUNX2 and inhibited the expression of MMP9, which is involved in bone matrix degradation. Our network pharmacology analysis identified the PI3K/AKT/ERK signaling pathway as a potential mechanism of DHG, a prediction strongly corroborated by *in vitro* experiments. The results showed that P2 active peptide from DHG could activate the PI3K/AKT/ERK pathway in BMSCs, promoting the phosphorylation of AKT and ERK, which in turn upregulated RUNX2 expression, ultimately driving osteoblast differentiation and mineralization. This aligns with existing literature, which reports that collagen peptides can regulate the PI3K/AKT pathway to promote osteogenesis (Zhu et al., 2020b). The pro-osteogenic effect of P2 was significantly blocked by the PI3K inhibitor LY294002, thereby confirming the “PI3K/AKT/ERK” axis as the core signaling pathway for its bone-protective effects.

Collagen is the most abundant protein in animals (Li and Wu, 2018), providing structural support. Still, it also contains bioactive peptides with various physiological activities, including beneficial effects on bone (Fu et al., 2019). Previously, we identified species-specific peptide biomarkers in DHG (Liu et al., 2019). Studies on collagen peptides from sources other than DHG have demonstrated various beneficial cellular activities, including antioxidant and anti-inflammatory properties (Hijlkema et al., 2022; Xin et al., 2025), which are believed to mitigate chemotherapy-induced organ damage. Coupled with our discovery that DHG influences the PI3K/AKT/ERK key signaling pathway relevant to multiple tissues, this positions DHG as a promising candidate for broader investigation within the context of comprehensive cancer survivorship care. This study identified 10 anti-OP active peptides from DHG, representing the potential material basis for its pharmacological effects. However, their specific pharmacological activities still require further validation.

Our network pharmacology analysis revealed a significant overlap in the molecular targets of CVDs and OP, including AKT1, TP53, ALB, MYC, STAT3, TNF, and IL-6, etc., which are regulated by key signaling cascades such as PI3K-AKT and MAPK. Critically, these pathways are not only central to the pathophysiology of both CVDs and OP but also serve as primary therapeutic targets for numerous anticancer agents, including cyclophosphamide (Afshari et al., 2020; Li et al., 2021). Anticancer strategies typically rely on inducing DNA damage to activate pro-apoptotic pathways like those mediated by TP53 (Yamashita et al., 2022; Man et al., 2023), or inhibiting pro-survival signals such as the PI3K-AKT cascade to halt tumor proliferation (Estruch et al., 2021; Zhang et al., 2019). However, the systemic nature of these pathways means such interventions inevitably cause off-target effects. For example, chemotherapeutic agents such as anthracyclines induce systemic reactive oxygen species (ROS) and oxidative stress, which disrupt the basic PI3K/AKT and MAPK signaling pathways (Xie et al., 2024), leading to simultaneous damage to myocardial and bone cells (Wang et al., 2024; Narezkina et al., 2021). Additionally, the established “bone-heart axis” (Liu et al., 2025) further underscores this interconnection. Within this framework, DHG emerges as a potent modulator. Our findings demonstrate that DHG exerts its therapeutic effects by targeting these same PI3K/AKT/MAPK pathways. Given the established role of the PI3K/AKT pathway in protecting cardiomyocytes from chemotherapy-induced apoptosis and dysfunction (Campia et al., 2019), DHG’s ability to modulate this specific cascade provides strong evidence for its dual-protective role against both cardiac and skeletal damage. This mechanism is consistent with Traditional Chinese Medicine theory, where DHG is believed to “nourish the kidneys, generate blood, and strengthen tendons and bones” (Wu et al., 2020a; Guizhou Medical Products Administration, 2019). Moreover, the holistic improvements observed in DHG-treated rats, including enhanced physical resilience and mitigation of chemotherapy-related side effects, suggest its benefits transcend organ-specific protection, conferring broad systemic advantages during cancer therapy.

Despite our research providing valuable insights into the bone-protective effects of DHG during chemotherapy, the understanding of the multi-organ side effects induced by chemotherapy remains incomplete. Moreover, the parameters or histological changes in key organs outside the skeleton due to CTX or DHG have not been fully assessed. It is noteworthy that the PI3K/AKT pathway is a key dysregulated intracellular signaling pathway in many malignant tumors, promoting tumor cell proliferation and angiogenesis, inhibiting apoptosis, and is closely associated with tumor invasion and metastasis (He et al., 2021; Rascio et al., 2021). Therefore, in the presence of tumors, the bone-protective effect of DHG mediated by the PI3K/AKT/ERK pathway may conflict with the antitumor effects induced by chemotherapy. However, in clinical practice, collagen-based nutritional supplements are commonly administered to patients after completing chemotherapy to improve their quality of life (Wang et al., 2020; Wang et al., 2014). Additionally, studies show that the relevant formulation with modified Guilu Erxian glue decoction enhances the sensitivity of cisplatin in lung cancer models resistant to chemotherapeutic drugs, significantly improving the immune function of nude mice resistant to lung cancer (Shi et al., 2021). Future research is necessary to specifically study the effects of DHG and its bioactive components on the toxicity induced by chemotherapy in other related organs, as well as their roles in tumor models, to evaluate their potential applications in more complex clinical scenarios.

## 5 Conclusion

In conclusion, this study demonstrates that DHG, driven by its core functional peptides, effectively prevents chemotherapy-induced osteoporosis in rats. This protective effect is mediated through the modulation of the PI3K/AKT/ERK signaling pathway, which enhances Runx2 expression while reducing MMP9 levels, thereby promoting osteoblast function and bone formation. More significantly, by identifying this PI3K/AKT-dependent mechanism, our research reveals a common pathway in the “bone-heart axis” that is susceptible to chemotherapy-induced injury, providing a new theoretical framework for the application of DHG in cardio-oncology. While this exploratory investigation offers valuable insights for developing natural drugs that can systematically mitigate the side effects of chemotherapy, we acknowledge that substantial future work is necessary to verify the clinical safety and efficacy of DHG. Nevertheless, these findings strongly suggest that DHG and its mediated signaling pathways are promising directions for future research aimed at providing multi-organ protection in comprehensive cancer survivorship care.

## Data Availability

The original contributions presented in the study are included in the article/Supplementary Material, further inquiries can be directed to the corresponding author.
