# Supplementary material for: Bone-protective effects of deer-hide gelatin in cyclophosphamide-induced osteoporosis rats
Source: Front Pharmacol. 2025 Oct 7;16:1631924. doi: 10.3389/fphar.2025.1631924 (PMC12537715; doi:10.3389/fphar.2025.1631924)
Supplement: Supplementary file 1 [file DataSheet1.docx]

Supplementary Material

# Supplementary Figures

#
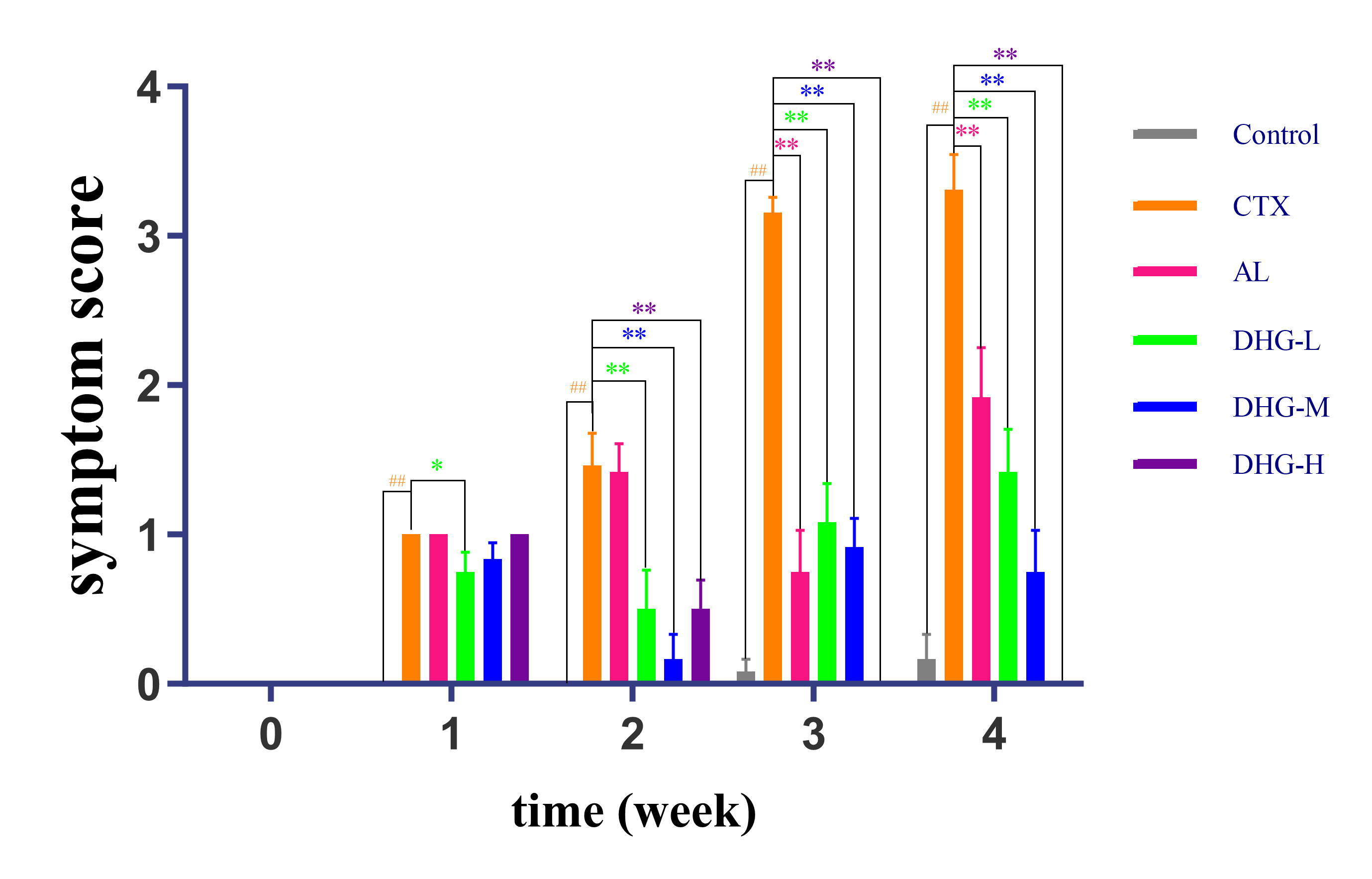


**Supplementary Figure 1. Effect of DHG on symptom scores during the weeks of CTX-induced OP rats.** Statistical significance was evaluated by one-way ANOVA, and multiple comparisons between groups were performed by the Dunnett test. Values are expressed as mean ± standard error of the mean (*n* = 11 to 12 rats per group). ^##^P < 0.01 vs Control in the same week; ^*^P < 0.05, ^**^P < 0.01 vs CTX in the same week. Abbreviations: CTX, cyclophosphamide; AL, alendronate (7.35 mg/kg); DHG-L, deer-hide gelatin low dose (0.27 g/kg); DHG-M, deer-hide gelatin medium dose (0.54 g/kg); DHG-H, deer-hide gelatin high dose (1.08 g/kg).

#
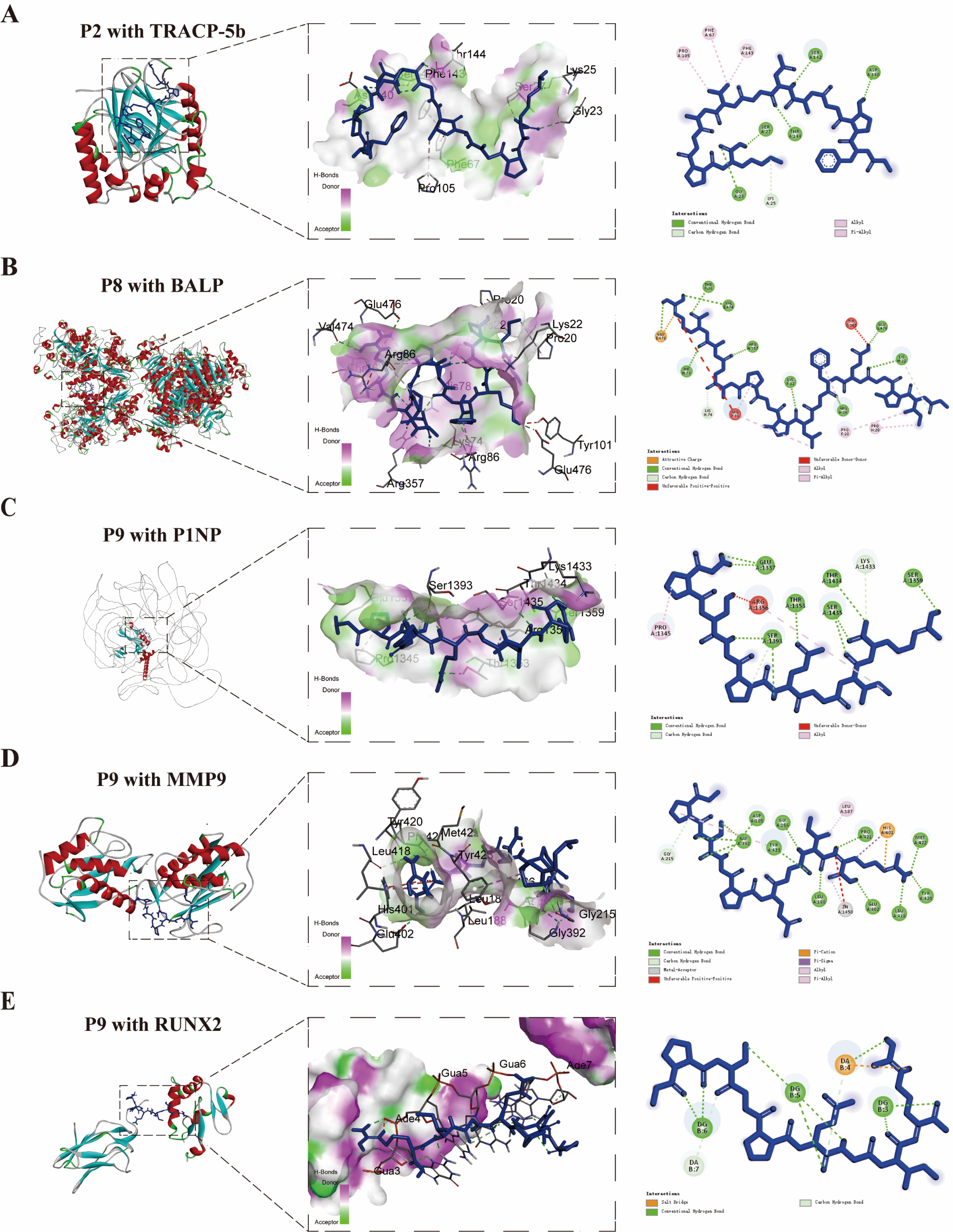


**Supplementary Figure 2. The outcomes of molecular docking between the DHG active peptides and targets.** Molecular docking results of (A) P2 and TRACP-5b, (B) P8 and BALP, (C) P9 and P1NP, (D) P9 and MMP9, (E) P9 and Runx2. TRACP-5b: tartrate-resistant acid phosphatase 5b; BALP: bone-specific alkaline phosphatase; P1NP: N-terminal propeptide of type I procollagen; MMP9: matrix metalloproteinase-9; Runx2: runt-related transcription factor 2.


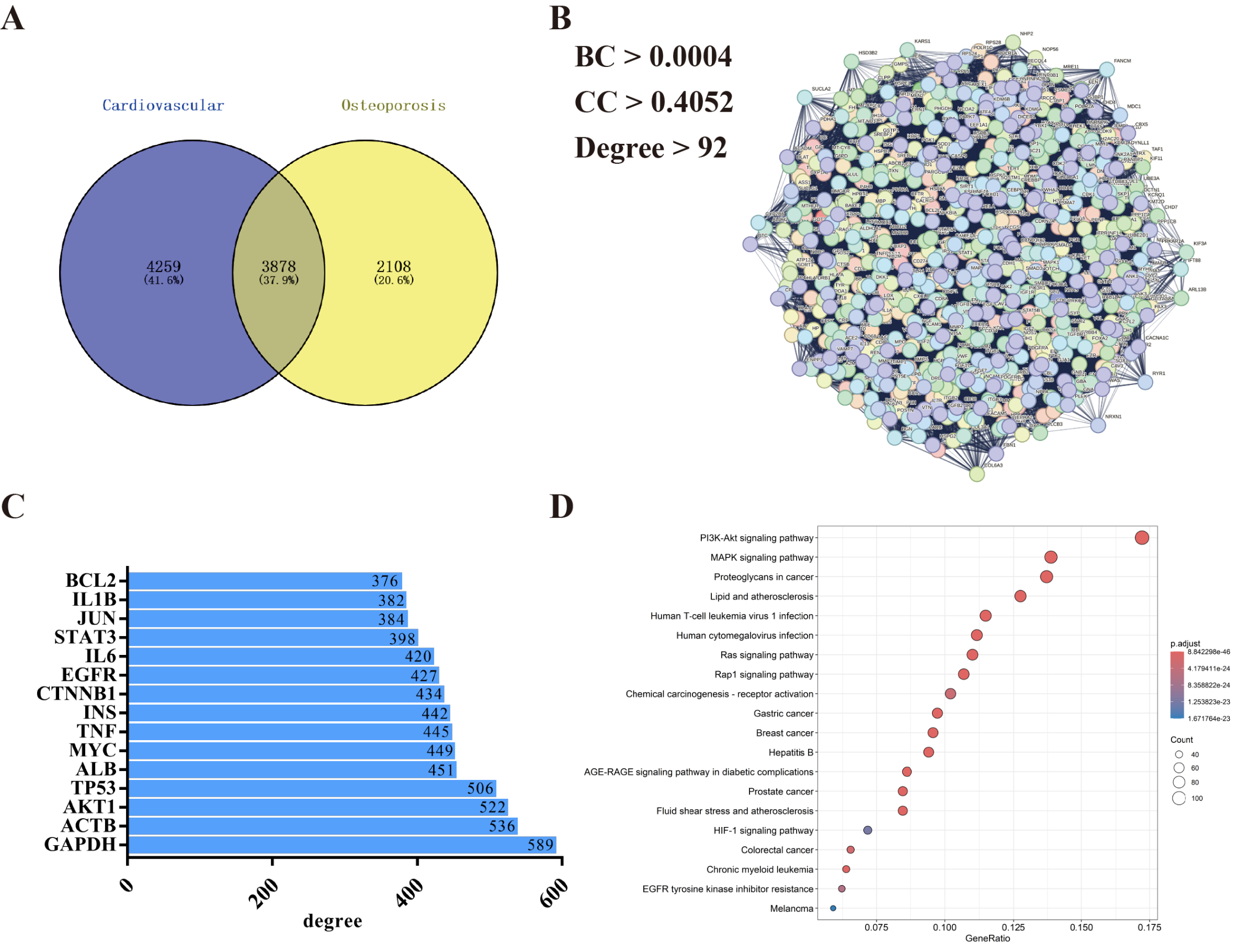


**Supplementary Figure 3. Network pharmacology analysis between CVDs and OP.** (A) Distribution of Cardiovascular diseases and OP target genes. (B) Protein-protein interactions network of 683 core targets of Cardiovascular diseases and OP. (C) Top 15 targets by degree in the PPI network. (D) Top 20 pathways for KEGG enrichment analysis.
